# The Use of Microporous Polysaccharide Hemospheres in Thyroid Surgery: A Retrospective Study on Safety and Clinical Outcomes

**DOI:** 10.3390/medicina61122209

**Published:** 2025-12-15

**Authors:** Cinzia Mariani, Amina Al Dababsekh, Filippo Carta, Mauro Bontempi, Carmelo Barbaccia, Roberto Puxeddu

**Affiliations:** 1 Unit of Otorhinolaryngology, Department of Surgery, Azienda Ospedaliero-Universitaria di Cagliari, University of Cagliari, 09100 Cagliari, Italy; cinzia.mariani@unica.it (C.M.);; 2 Unit of Otorhinolaryngology, Department of Surgery, King’s College Hospital London, Dubai P.O. Box 340901, United Arab Emirates; draminadababseh@gmail.com (A.A.D.);

**Keywords:** Arista, thyroidectomy, hemostasis, postoperative complications

## Abstract

*Background and Objectives*: Hemostasis during thyroid surgery is crucial to avoid postoperative complications, particularly hematoma, which can cause life-threatening airway compromise. Arista™ AH, a plant-based absorbable hemostatic powder, is designed to enhance intraoperative bleeding control. The present study evaluates the efficacy and safety of Arista™ AH in thyroidectomy. *Materials and Methods*: This retrospective study included 102 patients who underwent thyroidectomy between January 2020 and February 2024. Of these, 63 patients (61.8%) received Arista™ AH as an adjunctive hemostatic agent, and 39 (38.2%) underwent only conventional hemostasis. Outcomes measured included the incidence of postoperative hematoma and seroma, adverse events related to the use of Arista™ AH, and length of hospital stay. *Results*: None of the patients in the Arista™ AH group developed a hematoma in the thyroid surgical bed, whereas this complication occurred in 2 patients (5.1%) of the control group (*p* = 0.07). No patients in the Arista™ AH group developed a postoperative seroma, compared with 2 patients (5.1%) in the control group (*p* = 0.07). No adverse events related to Arista™ AH were reported. Length of hospital stay was similar between groups, with a median of 2 days (IQR 1) in both the Arista™ AH and control groups (*p* = 0.8). *Conclusions*: Arista™ AH was associated with favorable postoperative outcomes in thyroid surgery, supporting its safe and effective use as a hemostatic adjunct.

## 1. Introduction

Thyroidectomy remains a common procedure worldwide for both benign and malignant thyroid disease. Despite its routine nature, thyroid surgery carries specific risks due to the gland’s high vascularity and its proximity to critical anatomical structures.

Hypoparathyroidism, recurrent laryngeal nerve paralysis, loss of high-pitched voice, hematoma, and seroma are well-known complications of thyroidectomy [1]. Among these, hemorrhage and hematoma formation are considered the most feared postoperative complications [2], due to their potentially life-threatening nature if not promptly diagnosed and treated. Even small hematomas may result in patient distress, prolonged hospitalization, and increased health care costs. This risk is particularly relevant in modern surgical practice, where early discharge or outpatient thyroidectomy is increasingly adopted.

Post-thyroidectomy hematoma may present as hoarseness, dysphagia, and dyspnea, which can progress to complete airway obstruction. Clinical signs include progressive neck swelling, bleeding from the suture line, ecchymosis over the surgical site, and when the drain is left in situ, significant drainage output [3]. Hematomas typically occur within the first few hours after surgery, requiring urgent surgical re-intervention. The reported incidence ranges from 0.1% to 4.7%, depending on patient characteristics, comorbidities, and surgical technique [1,3,4]. According to current literature, several factors seem to be associated with an increased risk of hematoma formation following thyroid surgery, such as male sex, advanced age, hypertension, Graves’ disease, the use of anticoagulant therapy, prior thyroid surgery, total thyroidectomy, and neck dissection [5,6].

Achieving meticulous hemostasis during thyroidectomy is essential to prevent hemorrhagic complications. Traditional techniques, including electrocautery, energy-based instruments, vessel ligation, suturing, and surgical clips, are effective but may not fully control diffuse microvascular bleeding and venous oozing, conditions commonly encountered after thyroid gland mobilization. Moreover, certain anatomical areas within the thyroid bed present additional challenges for conventional hemostatic tools. In regions close to the recurrent laryngeal nerve or the parathyroid glands, the use of thermal instruments may increase the risk of thermal injury or devascularization.

For these reasons, adjunctive topical hemostatic agents have been increasingly used to complement conventional techniques. Their purpose is not to replace meticulous surgical hemostasis, but to optimize bleeding control and minimize postoperative complications. These agents can be divided into hemostats and surgical sealants, and can differ in terms of their materials, composition, methods of preparation, use and conservation [4]. Topical hemostatic powders have gained increasing popularity because they can be rapidly applied over large areas, cover irregular anatomical surfaces, and act independently of the patient’s coagulation status. Compared with gel-based or biologically active agents, powder hemostats offer easier handling, quicker activation, and lower preparation time [7,8].

Arista™ AH (Advanced Hemostat) is a sterile, plant-derived, absorbable hemostatic powder composed of microporous polysaccharide hemospheres (MPHs). It promotes hemostasis by absorbing water, concentrating clotting factors, and accelerating clot formation [2]. Compared to other hemostatic agents, it offers the benefit of rapid absorption, typically within 48 h, without leaving residues [8,9]. Its ease of use and favorable safety profile have led to widespread adoption across different surgical specialties [7,10,11], although evidence specifically supporting its use in thyroid surgery remains limited.

The purpose of this study is to evaluate the efficacy and safety of Arista™ AH in reducing postoperative hemorrhagic complications in patients who underwent thyroid surgery, compared with conventional hemostatic techniques.

## 2. Materials and Methods

A retrospective cohort study was conducted at the Unit of Otorhinolaryngology, King’s College Hospital London in Dubai, after obtaining approval from the institutional review board (IRB Approval No. KCH-REC-050).

### 2.1. Study Population

The study included all consecutive adult patients who underwent total or partial thyroidectomy between January 2020 and February 2024.

Inclusion criteria were:

- Age ≥ 18 years;
- Undergoing total or partial thyroidectomy for benign or malignant thyroid pathology;
- Availability of complete clinical, surgical, and postoperative data.

Exclusion criteria were:

- Known coagulopathy or other bleeding disorders;
- Ongoing anticoagulant therapy without appropriate perioperative reversal;
- Poorly controlled hypertension despite therapy;
- History of previous neck surgery;
- Incomplete or missing medical records.

Data, including demographics, surgical details, and postoperative outcomes, were extracted from electronic medical records and operative notes. Patients with missing or incomplete data were excluded from the final analysis.

Since February 2022, surgeons routinely adopted the use of the Arista™ AH (manufactured by Becton, Dickinson and Company (BD), Franklin Lakes, NJ, USA) powder as an adjunctive hemostatic agent in clinical practice.

Based on the type of hemostasis received, patients were categorized into two groups. The Arista™ AH group (n = 63) received adjunctive Arista™ AH powder (5 g) following thyroid resection, while the control group (n = 39) underwent only conventional hemostasis using absorbable sutures and the LigaSure™ vessel-sealing system (Medtronic, Minneapolis, MN, USA), which was the standard energy device available in our department throughout the study period.

### 2.2. Preoperative Evaluation and Surgical Technique

All patients underwent standardized preoperative evaluation, including thyroid function tests, serum calcium levels, ultrasound examination of the neck, fine needle cytology (FNC) of the thyroid lesion, and fiberoptic laryngoscopy within 30 days prior to surgery.

Indications for surgery included large and/or symptomatic benign goiters, Graves’ disease, and thyroid cancer (FNC-suspicious or malignant lesions).

All surgeries over the study period were performed by two experienced head and neck surgeons.

Additionally, there were no major changes in surgical technique or protocol throughout the study period.

All procedures were performed under general anesthesia with endotracheal intubation.

Intraoperative nerve monitoring (IONM) was routinely used to facilitate nerve identification and confirm functional integrity throughout the procedure. A standard Kocher incision was made along a natural skin crease, followed by elevation of subplatysmal flaps. The midline raphe of the strap muscles was divided to expose the thyroid gland. The superior and inferior thyroid pedicles were dissected using a combination of ligation, bipolar cautery, and LigaSure™ vessel-sealing system. Particular attention was paid to preserving the external branch of the superior laryngeal nerve and the parathyroid glands.

**Figure 1:**
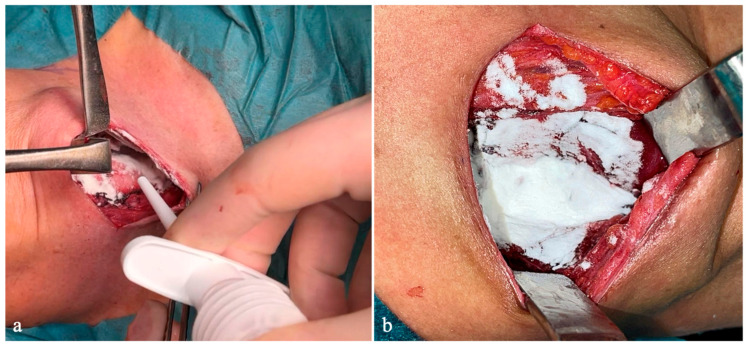
Surgical field following thyroidectomy before (**a**) and after (**b**) hemostatic application.

In patients in the Arista™ AH group, the hemostatic powder was applied immediately after completion of thyroid resection and before wound closure. A 5 g dose was evenly distributed over the thyroid bed, tracheal surface, and areas of potential venous oozing (Figure 1). Suction was minimized during and after application to prevent removal of the powder before clot formation occurred.

A surgical drain was generally placed, particularly in cases of extensive dissection fields, difficult hemostasis, or concomitant neck dissection.

### 2.3. Study Outcomes

The study assessed both primary and secondary outcomes.

The primary outcome was the incidence of postoperative hematoma requiring surgical drainage.

Secondary outcomes included the occurrence of seroma formation, any adverse events related to the use of Arista™ AH, and the length of hospital stay.

Other common postoperative complications of thyroid surgery, such as transient hypocalcemia or recurrent laryngeal nerve injury, were not included in the analysis because they are unrelated to the use of adjunctive hemostatic agents.

### 2.4. Statistical Analysis

Continuous variables (age, operative time, and length of hospital stay) were first assessed for distribution using the Shapiro–Wilk test. Age demonstrated a normal distribution, whereas operative time and length of stay deviated significantly from normality. Therefore, age was analyzed using Student’s *t*-test and presented as mean ± standard deviation. Because operative time and length of hospital stay were not normally distributed according to the Shapiro–Wilk test, these variables were analyzed using the Mann–Whitney U test and are reported as median and interquartile range (IQR), which represent the appropriate measures of central tendency and dispersion for non-normally distributed data. Categorical variables were analyzed using Chi-square test or Fisher’s exact test, as appropriate. A *p*-value < 0.05 was considered statistically significant. All analyses were performed using SPSS version 25.0 (IBM Corp., Armonk, NY, USA).

## 3. Results

A total of 102 patients (21 males, 81 females; mean age 43.6 years, range 18-75 years) underwent thyroidectomy between January 2020 and February 2024. Among them, 63 patients (61.8%) received Arista™ AH as an adjunctive hemostatic agent, while 39 patients (38.2%) underwent surgery using conventional hemostasis with absorbable sutures, electrocautery, and the LigaSure™ vessel-sealing system.

There were no statistically significant differences in baseline characteristics between the groups in terms of age, gender, and surgical approach (total vs. partial thyroidectomy), as shown in Table 1.

During the postoperative period, a total of three patients required surgical drainage for bleeding: one patient in the Arista™ AH group, who developed bleeding outside the thyroid surgical bed, and 2 of 39 patients (5.1%) in the control group, who developed postoperative hematoma in the thyroid surgical bed. Both patients in the control group had undergone total thyroidectomy and neck dissection for thyroid carcinoma. In the only patient in the Arista™ AH group who experienced bleeding, the source was identified as an anterior jugular vein located anterior to the strap muscles and unrelated to the thyroid surgical bed where Arista™ AH had been applied. None of the patients who developed hematoma required a tracheotomy. There were no deaths, and no patients required blood transfusion.

None of the patients in the Arista™ AH group developed a postoperative seroma, whereas seroma occurred in 2 patients (5.1%) in the control group (*p* = 0.07), requiring repeated outpatient needle aspiration.

No wound infections were observed in any of the patients in either group.

No adverse events related to Arista™ AH were reported in any patient.

Length of hospital stay was similar between groups, with a median of 2 days (IQR 1) in both the Arista™ AH and control groups. The Mann–Whitney U test confirmed no statistically significant difference (*p* = 0.8).

## 4. Discussion

Effective hemostasis during thyroid surgery is essential to minimize the risk of postoperative complications such as hematoma, seroma formation, and prolonged hospital stays.

Arista™ AH, a MPH derived from purified potato starch, has emerged as a widely used absorbable hemostatic powder due to its broad surface coverage, rapid absorption, and safety profile. It is classified as a mechanical hemostat. These products function by rapidly absorbing fluid at the bleeding surface, thereby concentrating clotting components and supporting the formation of a stable hemostatic plug [4,8]. Mechanical hemostats are commonly used during surgery to manage diffuse bleeding, areas that are difficult to access, and persistent oozing from raw surfaces [2,8].

Upon contact, Arista™ AH immediately starts dehydrating the blood to form a gelled matrix. The osmotic action concentrates serum proteins, platelets, and other clotting factors on the surface of the Arista™ AH, creating a scaffold for the formation of a strong fibrin clot within a few minutes from application [8]. The product is fully absorbed within 24–48 h, thereby avoiding complications associated with non-resorbable materials [4].

Several studies have already assessed the efficacy and safety of Arista™ AH in various surgical procedures, including cardiac, head and neck, and general surgeries [2,7,10,11].

A retrospective study involving 240 patients who underwent cardiothoracic surgery demonstrated that Arista™ AH significantly reduced hemostasis time, postoperative chest tube output during the first 48 h, and the need for postoperative blood transfusion [7].

An observational study on patients who underwent sinus surgery showed that Arista™ AH effectively controlled bleeding, achieving hemostasis within approximately 30–45 seconds after application [10]. Promising results were also observed in a randomized, controlled study of 40 patients who underwent nasal surgery, showing that Arista™ AH significantly reduced postoperative bleeding compared to the control group, with no notable differences in the incidence of infections, edema, synechiae development, pain, obstruction, or nasal discharge between the groups [11].

In thyroid surgery specifically, only Kunduz et al. [2] have conducted a randomized controlled trial evaluating the hemostatic efficacy of Arista™ AH, compared with conventional hemostatic techniques and another hemostatic powder (Haemocer^TM^). Although no statistically significant reduction in postoperative drainage or complications was observed, the use of Arista™ AH was associated with a trend toward lower drainage volumes compared with conventional hemostatic techniques.

Consistent with the findings of Kunduz et al. [2], we did not demonstrate a statistically significant superiority of Arista™ AH over conventional hemostasis, likely due to the low incidence of postoperative bleeding and the limited sample size. However, the consistent absence of hematoma and seroma in the Arista™ AH group compared with the control group suggests a clinically favorable trend. Notably, none of the patients in the Arista™ AH group developed a hematoma in the thyroid surgical bed, whereas this complication occurred in 2 of the 39 patients (5.1%) in the control group (*p* = 0.07), both of whom required surgical drainage. Both postoperative hematomas occurred in patients who had undergone total thyroidectomy with associated neck dissection for thyroid carcinoma. This observation aligns with previous reports suggesting that more extensive surgical procedures, particularly those involving lymph node dissection, carry a higher risk of postoperative bleeding due to the wider dissection area and greater vascular exposure [12,13]. However, it is noteworthy that no hematomas of the surgical thyroid bed occurred among patients who underwent total thyroidectomy with neck dissection and received Arista™ AH. This finding may indicate that the use of Arista™ AH provides additional intraoperative hemostatic control even in higher-risk surgical settings.

In the present study, all thyroidectomies were performed using the LigaSure™ vessel sealing system, which has been widely adopted for thyroid surgery due to its reliability in sealing vessels up to 7 mm in diameter and minimizing thermal spread to adjacent structures. Several studies have compared LigaSure™ with the Harmonic Scalpel (HS), showing that both devices offer comparable safety profiles—particularly in preventing major bleeding complications requiring reoperation—although HS has been reported to achieve slightly faster hemostasis in selected subgroups, such as patients with thyroid carcinoma [14,15].

Another common complication that may occur after thyroid surgery is seroma formation in the surgical bed. None of the patients in the Arista™ AH group developed a seroma. The lower, though not statistically significant, incidence of postoperative seroma in the Arista™ AH group compared to the control group (5.1%) (*p* = 0.07) suggests that Arista™ AH may contribute to reduced lymphatic leakage. Some authors [16] have highlighted the importance of minimizing tissue trauma and fluid accumulation in preventing seroma, objectives that Arista™ AH may support by promoting rapid coagulation and sealing of lymphovascular spaces.

The improved intraoperative hemostatic control achieved with Arista™ AH likely facilitated earlier drain removal and may have facilitated a stable postoperative course, although no difference in the length of hospital stay was demonstrated between groups. In high-volume surgical centers, lower rates of postoperative complications, could translate into cumulative benefits in terms of bed availability, workflow efficiency, and overall resource utilization.

Importantly, no adverse reactions to Arista™ AH were reported in this study, consistent with the findings of LyBarger et al. [8], who emphasized its biocompatibility and minimal inflammatory response.

Although Arista™ AH may not provide a measurable improvement in hemostatic efficacy compared with other topical agents, its primary advantages lie in its excellent safety profile and ease of use. Its plant-derived, biocompatible composition allows for broad surface coverage and uniform distribution, while its rapid absorption within 24–48 h prevents the formation of residual material that could otherwise mimic thyroid remnants or lymphatic fluid collections on postoperative imaging. Delayed resorption of hemostatic agents, in fact, can lead to diagnostic errors that may affect the patient’s therapeutic pathway, potentially being mistaken for residual thyroid tissue in the surgical bed during follow-up imaging [17]. Moreover, the simple and controlled application process facilitates efficient intraoperative workflow, as demonstrated by the shorter operative time in the Arista™ AH group, and may reduce surgeon stress during diffuse or low-volume bleeding.

A unique safety consideration with this hemostatic agent, derived from purified potato starch, is the recommendation to limit its use to no more than 50 g in diabetic patients to prevent elevated postoperative glucose levels [9].

The main strengths of this study include the homogeneity of the surgical technique, as all procedures were performed by two experienced head and neck surgeons using standardized protocols throughout the study period. Furthermore, the inclusion of consecutive patients and the availability of complete clinical and surgical data minimized selection bias and ensured the reliability of the findings. Another notable strength is the real-world design, reflecting routine surgical practice rather than a highly selective experimental setting. This enhances the generalizability of our results and provides clinically meaningful insight into the practical use of Arista™ AH in thyroid surgery.

Limitations of the study include its retrospective design, single-center scope, and relatively small patient cohort. A post hoc power analysis showed that, given the sample sizes of the two groups (63 vs. 39 patients) and an observed hematoma and seroma rate of ~5% in the control group, the study had limited statistical power (<30%) to detect a 5% absolute reduction. The absence of statistical significance is likely attributable to insufficient sample size rather than a true lack of effect, and the favorable clinical trends observed warrant evaluation in larger, prospective, and adequately powered studies.

The systematic use of Arista™ AH could impact costs, with an average cost of approximately 120 dollars per surgery in our experience. Although this represents an added expense, it remains substantially lower than that of many biologically active or dual-component hemostatic agents, such as fibrin sealants, as reported by Allotey et al. in their systematic review [18]. Moreover, this added cost it may be offset if the product helps reduce operative time or postoperative complications, as observed in our series, since these factors are major determinants of total healthcare expenditure. Until larger prospective studies confirm these potential benefits, its use may be best reserved for patients at increased risk of bleeding. A prospective, randomized, multicenter controlled trial with a larger sample size and an integrated cost-effectiveness analysis would provide stronger evidence for the broader adoption of Arista™ AH in thyroid surgery.

## 5. Conclusions

The results of this study support the use of Arista™ AH as a safe and effective adjunctive hemostatic agent in thyroid surgery. Its application was associated with improved hemostatic control and fewer postoperative complications. Although larger prospective studies are needed to validate these findings and assess the long-term cost-effectiveness of its routine use, the present results suggest that Arista™ AH represents a valuable tool for optimizing safety and efficiency in thyroidectomy.

## Figures and Tables

**Table 1:** Demographic and baseline characteristics of the study cohort.

Variable	Control Group (n = 39)	Arista™ AH Group (n = 63)	*p* Value
Mean age (years)	44.2 ± 11.1	42.9 ± 10.6	0.6
Gender (F/M)	30/9	51/12	0.6
Benign thyroid pathology	20 (51.3%)	43 (68.3%)	0.09
Malignant thyroid pathology	19 (48.7%)	20 (31.7%)	0.09
Total thyroidectomy	24 (61.5%)	37 (58.7%)	0.8
Partial thyroidectomy	15 (38.5%)	26 (41.3%)	0.8
Median Operative Time (min)	82 (IQR 46.5)	75 (IQR 40.5)	<0.05

IQR = interquartile range.

## Data Availability

The data presented in this study are available on request from the corresponding author.
